# The prognostic value of retinol binding protein in geriatric hip fractures after surgeries: A propensity score matching and 1-year follow-up study

**DOI:** 10.1186/s12891-022-06068-0

**Published:** 2022-12-12

**Authors:** Mingchong Liu, Chensong Yang, Xian Xu, Shengchao Ji, Yutao Pan, Ning Han, Shimin Chang, Guixin Sun

**Affiliations:** 1 Department of Traumatic Surgery, School of Medicine, Shanghai East Hospital, Tongji University, Shanghai, 200092 China; 2 Yangpu Hospital, School of Medicine, Tongji University, Shanghai, 200092 China

**Keywords:** Hip fracture, Retinol binding protein, Prognosis, Hip surgery, PSM study

## Abstract

**Background:**

We aimed to explore the predictive value of retinol binding protein (RBP) for outcomes of hip fractures.

**Methods:**

Patients with hip fractures who underwent hip surgeries between December 2017 and February 2021 and met the inclusion criteria were analyzed. Propensity score matching was used to reduce the bias of co-factors and ROC curves based on matched populations were created to determine the optimal cutoff point of RBP. The outcomes between patients with low levels of RBP and high levels of RBP were compared.

**Results:**

Four hundred eighty patients were enrolled in this study and 69 patients died within one year. After a 1:1 PSM, patients with more than 1-year survival had significantly higher RBP (*p = *0.013) than those who died within one year, as well as patients divided by 6-months survival (*p* = 0.012). Logistics analysis showed that low RBP may be an independent risk factor for 3-month survival, 6-month survival, 1-year survival, and 3-month free walking ability.

**Conclusion:**

RBP may be associated with the survival and 3-month walking abilities of patients with hip fractures.

## Introduction

Hip fracture is one of the most severe and frequently-occurring fractures in middle-aged and older people [1]. Hip fractures always occur in individuals with osteoporosis, mostly caused by falls [2]. Patients with hip fractures often face obvious pain and disability, so surgeries were required to reduce the duration of pain and avoid prolonged bed stays [3]. However, older patients tend to meet a variety of comorbidities and complicated medical conditions such as coronary heart disease, hypertension, diabetes, and chronic obstructive pulmonary disease, and those patients need to stay in bed for a long time, even after surgery [4]. Unable to walk and a long stay in bed could cause multiple complications, such as hypostatic pneumonia, deep vein thrombosis, bedsore, urinary infection, and so on, and these are often the leading causes of death for patients with hip fractures [5].

Many studies had proved that bone regeneration activities and nutrition status may affect the rehabilitation and outcomes after surgeries for patients with fractures [6, 7]. Bone regeneration was regulated by two types of cells: the osteoblasts, which involve in bone forming, and the osteoclasts, which regulate bone resorption [8]. Strong bone regeneration abilities can accelerate bone healing and bone matrix regeneration by facilitating osteoblasts, and then the patients with strong bone metabolism may get up earlier and face fewer complications [9]. Nutrition is also an important factor for hip rehabilitation: favorable nutrition status could not only provide a sufficient foundation for bone healing but also reduce the potential infectious risk for patients after surgeries [10]. Therefore, an optimal marker that may indicate the status of bone metabolism and body nutrition may predict the outcome of hip fractures after surgery and then can provide more proven information for clinical practice and rehabilitation of patients.

Retinol binding protein (RBP) is a group of proteins responsible for the binding and transport of retinol active metabolites. RBP is synthesized by the liver and widely distributed in the blood, cerebrospinal fluid, and urine [11]. It is reported that RBP was positively associated with bone mineral density (BMD) and the retinol binding protein 4 (RBP4) may also affect the formation of chondrocytes [12, 13]. At the same time, RBP is also used as a sensitive evaluation index of clinical nutritional status for the diagnosis of early malnutrition [14]. RBP, which may associate with bone metabolism and nutrition, might be a potential marker to predict the rehabilitation and outcomes of patients who underwent hip surgeries due to hip fractures, and few studies reported the RBP and the prognosis of hip fracture. Therefore, we conducted this study to explore the relationship between RBP and hip fractures. 

## Material and methods

### Study design and participants

This is a retrospective observational study conducted at the Department of Traumatology, Shanghai East Hospital, Tongji University, School of Medicine, Shanghai, China. The study was approved by the Ethics Committee of East Hospital, and all the data concerned about patient privacy were well protected. Participants of our study were patients with hip fractures admitted to our department between December 2017 and February 2021. The data of patients who met the inclusion criteria were extracted from our database. The inclusion criteria were set as below: (1) surgeries performed for hip fractures; (2) age ≥ 50 years; (3) the fractures caused by low-energy, not high-energy trauma; (4) not pathological fractures; (5) patients without a diagnosis of severe liver diseases. The study was carried out according to the Declaration of Helsinki and written informed consent was obtained from all patients in this study.

### Variables

Demographic characteristics of participants enrolled in this study were retrieved and summarized: age, sex, body mass index (BMI), residence, side of the fracture, type of fracture, fracture history, smoking and alcoholism status, polytrauma, type of surgery, anesthesia, time from injury to surgery and so on. The types of surgery were summarized as internal fixation and arthroplasty. The data from auxiliary examinations and laboratory tests when the patient was admitted to our hospital were also extracted. The electrocardiogram and chest radiograph were reviewed by the senior author (Guixin, Sun) and those clinically significant were identified as “abnormal”. Comorbidities of patients including diabetes, circulatory diseases (hypertension, coronary heart disease), chronic obstructive pulmonary disease, prior stroke, dementia, Parkinson’s disease, digestive system disorders, chronic renal failure, rheumatologic disease, and osteoporosis were collected and used to calculate the Charlson comorbidity index (CCI) [15].

### Outcomes

The primary outcomes in this study were survival at 3 months, 6 months, and 1 year. The secondary outcomes were the free walking ability at 3 months, 6 months, and 1 year, the hospitalization cost, and the hospital stays. The free walking ability was identified as the status that patients could independently perform their daily activities, including eating, dressing, bathing, and shopping. All patients were followed up for one year to collect their survival and mobility status. The data of patients who came to our outpatient for review were collected by outpatient doctors and others were contacted by telephone. Survival time in our study was identified as the time from surgery to all-caused death and those who survived more than one year were defined as censored data.

### Statistical analyses

Continuous variables were expressed as mean ± standard deviation while categorical data were presented as count (percent). Independent Student’s T-tests were used for normally distributed data while Wilcoxon rank-sum tests were used for non-normally distributed data. Categorical variables were evaluated by the chi-square test or Fisher’s exact test. Baseline characteristics were compared and summarized. Then 1:1 propensity score matching (PSM) with a caliper of 0.2 in R software was performed between patients grouped by 6-month survival, 1-year survival, 6-month free walking ability, and 1-year free walking ability, respectively. The RBP in the matched groups was compared and ROC curves were established to identify the optimal cutoff points of RBP.

Four Cox models were established for continuous RBP and binary RBP and adjusted differently to prove the risk value of RBP for 1-year survival. Model 1 and Model 3 were adjusted for age, gout, hypertension, ALB, Hb, and CT. Model 2 and Model 4 were fully adjusted. Binary RBP was analyzed in Model 1 and Model 2 while continuous RBP was included in Model 3 and Model 4. Model 1 and Model 3 were direct entry models while Model 2 and Model 4 were conditional stepwise forward models. Kaplan–Meier and Log-rank tests were also performed to analyze the relationship between RBP and 1-year survival. To better prove the predictive value of RBP, Logistics regression was used. All *p* < 0.05 were considered statistically significant and statistical analyses were performed using SPSS version 26.0 (IBM, Armonk, NY, USA), GraphPad Prism version 8.0.1 (GraphPad Software San Diego, USA), and R software version 4.1.1 (R Foundation for Statistical Computing, Vienna, Austria).

## Results

### Study population

**Fig. 1:**
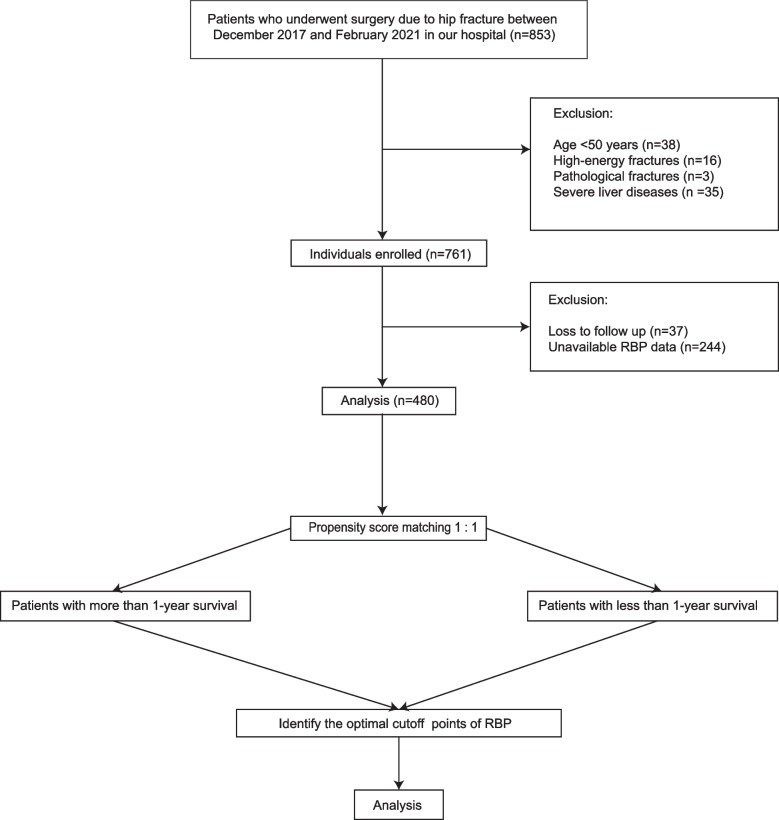
Flowcharts of our study

Eight hundred fifty three patients underwent hip surgeries in our department due to hip fractures from December 2017 to February 2021. 92 patients did not meet the inclusion criteria and 281 patients were excluded because of the unavailable RBP data and loss to follow up. Finally, 480 patients were enrolled in this study (Fig. 1). All the surgeries were performed by the senior author (Guixin, Sun) or in his presence and direction. The baseline features of patients grouped by 1-year survival status were shown in Table 1. The sex, chest radiograph, gout, hypertension, Hb, ALB, and RBP were significantly different between the two groups.

**Fig. 2:**
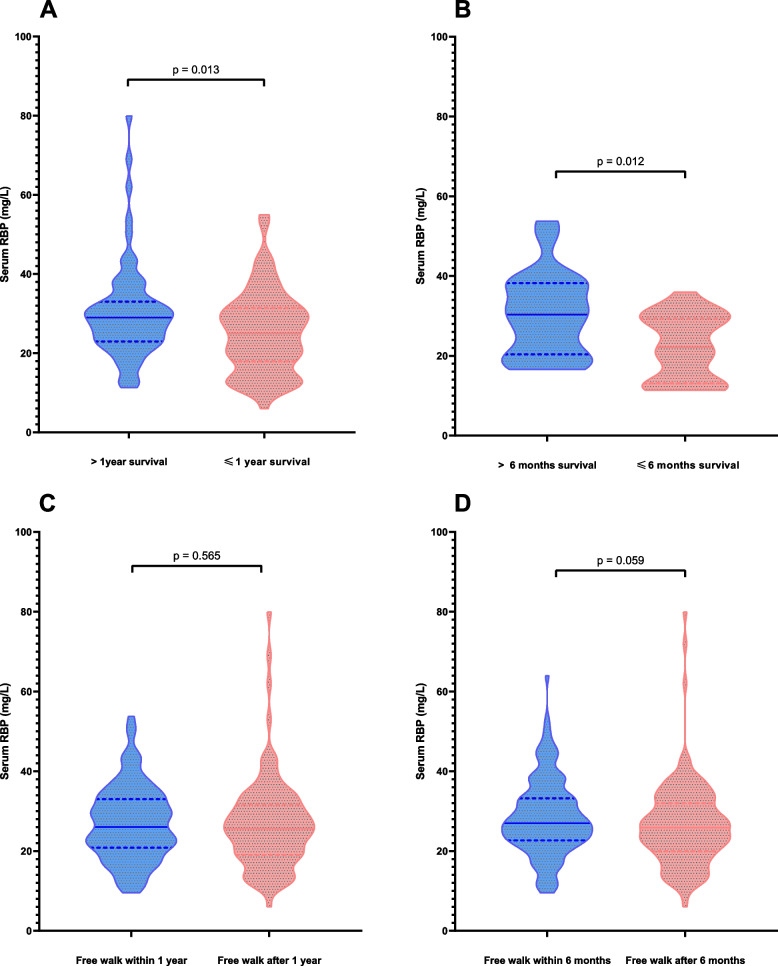
Serum levels of SOD and GR in the patients with hip fracture after surgery grouped by one-year survival (A/B), and ROC curves of SOD and GR for one-year survival (C/D). A/B: The patients who survived more than one year had significantly higher SOD levels (*p* = 0.026) than those who survived less than one year in the PSM population, as well as GR (*p* = 0.021); C/D: Both SOD (AUROC: 0.635) and GR (AUROC: 0.640) were able to predict the 1-year survival according to the ROC curves

**Table 1:** Baseline characteristics of patients grouped by the status of 1-year survival for unmatched and PSM population

Variables	Total (*n* = 480)	Unmatched population			Matched population		
		**Survival ≤ 1 year** (*n* = 69)	**Survival > 1 year** (*n* = 411)	*P*-value	**Survival ≤ 1 year** (*n* = 69)	**Survival > 1 year** (*n* = 69)	*P*-value
**Demographic characteristics**							
** Sex(female)**	**330 (68.8%)**	**51 (73.9%)**	**279 (67.9%)**	**0.317**	**51 (73.9%)**	**53 (76.8%)**	**0.693**
** Age(years)**	**74.93 ± 10.10**	**82.78 ± 7.49**	**73.62 ± 20.16**	** < 0.001**	**82.78 ± 7.49**	**81.65 ± 9.11**	**0.343**
** BMI (kg/m2)**	**23.00 ± 3.88**	**23.31 ± 3.32**	**22.94 ± 3.97**	**0.490**	**23.31 ± 3.32**	**23.22 ± 4.13**	**0.845**
** Residence (rural)**	**26 (5.4%)**	**3 (4.3%)**	**23 (5.6%)**	**0.891**	**3 (4.3%)**	**4 (5.8%)**	** > 0.999**
** Fractures history (yes)**	**80 (16.7%)**	**13 (18.8%)**	**67 (16.3%)**	**0.601**	**13 (18.8%)**	**11 (15.9%)**	**0.653**
** Smoking history (yes)**	**41 (8.5%)**	**5 (7.2%)**	**36 (8.8%)**	**0.677**	**5 (7.2%)**	**8 (11.6%)**	**0.382**
** Alcoholism history(yes)**	**15 (3.1%)**	**3 (4.3%)**	**12 (2.9%)**	**0.797**	**3 (4.3%)**	**3 (4.3%)**	** > 0.999**
** Position of fracture(right)**	**223 (46.5%)**	**29 (42.0%)**	**194 (47.2%)**	**0.425**	**29 (42.0%)**	**25 (36.2%)**	**0.485**
**Surgery-related variables**							
** Location of fracture(transcervical)**	**236 (49.2%)**	**29 (42.0%)**	**207 (50.4%)**	**0.200**	**29 (42.0%)**	**29 (42.0%)**	** > 0.999**
** Surgical procedures(arthroplasty)**	**177 (36.9%)**	**29 (42.0%)**	**148 (36.0%)**	**0.338**	**29 (42.0%)**	**30 (43.5%)**	**0.863**
** Anesthesia (spinal)**	**4 (0.8%)**	**1 (1.4%)**	**3 (0.7%)**	**0.464**	**1 (1.4%)**	**1 (1.4%)**	** > 0.999**
** Time from injury to surgery (Days)**	**5.74 ± 6.11**	**6.16 ± 5.00**	**5.67 ± 6.28**	**0.135**	**6.16 ± 5.00**	**5.78 ± 3.33**	**0.955**
** CCI score (> 4)**	**99 (20.6%)**	**20 (29.0%)**	**79 (29.2%)**	**0.064**	**20 (29.0%)**	**21 (30.4%)**	**0.852**
** Electrocardiogram (abnormal)**	**278 (57.9%)**	**42 (60.9%)**	**236 (57.4%)**	**0.591**	**42 (60.9%)**	**45 (65.2%)**	**0.597**
** Chest radiograph (abnormal)**	**228 (48.1%)**	**41 (59.4%)**	**187 (45.5%)**	**0.032**	**41 (59.4%)**	**40 (58.0%)**	**0.863**
** Gout (yes)**	**77 (16.0%)**	**29 (42.0%)**	**48 (11.7%)**	** < 0.001**	**29 (42.0%)**	**28 (40.6%)**	**0.863**
** Hypertension(yes)**	**239 (49.8%)**	**47 (68.1%)**	**192 (46.7%)**	**0.001**	**47 (58.1%)**	**46 (66.7%)**	**0.856**
** Polytrauma(yes)**	**67 (14.0%)**	**9 (13.0%)**	**58 (14.1%)**	**0.813**	**9 (13.0%)**	**7 (10.1%)**	**0.595**
**Laboratory findings**							
** Hb (g/L)**	**115.11 ± 20.66**	**109.51 ± 22.77**	**116.06 ± 20.16**	**0.005**	**109.51 ± 22.77**	**112.64 ± 20.21**	**0.310**
** INR**	**1.04 ± 0.12**	**1.04 ± 0.07**	**1.04 ± 0.12**	**0.287**	**1.04 ± 0.07**	**1.05 ± 0.13**	**0.524**
** GLU (mmol/L)**	**6.93 ± 3.55**	**7.04 ± 2.90**	**6.92 ± 3.65**	**0.674**	**7.04 ± 2.90**	**7.53 ± 6.39**	**0.623**
** ALB (g/L)**	**38.32 ± 4.34**	**37.01 ± 4.47**	**38.54 ± 4.25**	**0.008**	**37.01 ± 4.67**	**37.23 ± 3.60**	**0.716**
** UA (umol/l)**	**265.13 ± 87.56**	**273.79 ± 119.07**	**263.67 ± 81.18**	**0.975**	**273.79 ± 119.07**	**308.73 ± 108.88**	**0.060**
** RBP (mg/L)**	**27.73 ± 13.42**	**25.17 ± 10.31**	**28.41 ± 10.41**	**0.018**	**25.17 ± 10.32**	**30.20 ± 12.08**	**0.013**

Continuous variables were expressed as mean ± standard deviation and categorical variables were presented as count (percent). *BMI* body mass index, *Hb* Hemoglobin, *INR* International normalized ratio, *GLU* Blood glucose, *ALB* Albumin, *RBP* Retinol binding protein

### PSM and cutoff points of RBP

The baseline features of matched populations were summarized in Table 1. The levels of RBP were significantly higher in the patients with more than 1-year survival than those with less than 1-year survival (*p* = 0.013) in the matched study. Similarly, patients who survived more than 6 months had significantly high RBP than those who survived less than 6 months (*p* = 0.012) in the matched study (Fig. 2). ROC curves were established for 1-year survival and the area under the ROC curve were 0.635 (*p* = 0.026). The cutoff point of RBP was determined as 20.95 mg/L, whose Youden Index was the highest. Then the patients were grouped by high levels of RBP (> 20.95 mg/L) and low levels of RBP (≤ 20.95 mg/L).

### Relation between RBP and outcomes

**Fig. 3:**
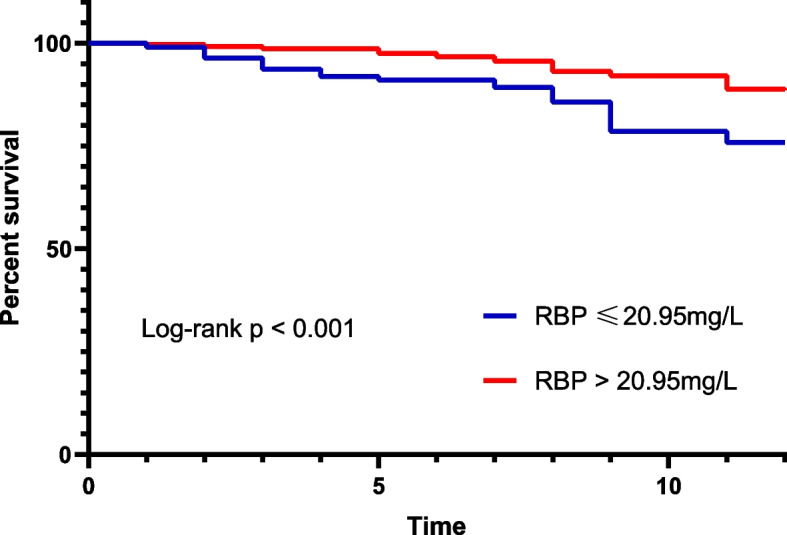
Kaplan–Meier analyses of one-year survival based on optimal serum levels of SOD (112.5 U/mL) and GR (52.5 U/L). A: Kaplan–Meier analysis showed that SOD values of 112.5 U/mL significantly differentiated patients who died within one year and survived more than one year (*p* < 0.001), B: SOD values of 52.5 U/L significantly differentiated patients who died within one year, and survived more than one year (*p* < 0.001)

Kaplan–Meier and Log-rank tests of RBP were shown in Fig. 3. Patients with high levels of RBP may have a higher survival probability than those with low levels of RBP (*p* < 0.001, Fig. 3). Similarly, cox models had shown that the RBP was a risk factor for death after hip surgery (Table 2). For binary RBP, patients with low levels of RBP may have a 175.0% increase in death risk (HR = 2.750, CI: 1.633–4.632) in Model 1 and a 150.1% increase (HR = 2.501, CI: 1.520–4.113) in Model 2. For continuous RBP, the increasing RBP may be a protective factor for survival in Model 3 (HR = 0.950, CI: 0.925–0.976) and in Model 4 (HR = 0.953, CI: 0.929–0.978). Outcomes of the low RBP population and high RBP population were compared and summarized in Table 3. The low RBP group may face poor outcomes than the high RBP group. To further prove the predictive value of RBP for survival and free walking ability, the logistics regression was performed for mortality and free walking ability at 3 months, 6 months, and 1 year. The low RBP group may have poor mortality at 3 months, 6 months, and 1 year, and free walking ability at 3 months, while for the continuous RBP, the increasing RBP may face favorable survival status at 3 months, 6 months, and 1 year (Table 4).

**Table 2:** Cox models of 1-year survival adjusted for different factors

Variables	Model 1		Model 2	
	**HR (95% CI)**	**p**	**HR (95% CI)**	**p**
**gout (yes)**	**5.258 (3.096, 8.929)**	** < 0.001**	**4.840 (2.950, 7,942)**	** < 0.001**
**Age (continuous)**	**1.108 (1.071, 1.146)**	** < 0.001**	**1.097 (1.065, 1.129)**	** < 0.001**
**RBP (≤ 20.95 mg/L)**	**2.750 (1.633, 4.632)**	** < 0.001**	**2.501 (1.520, 4.113)**	** < 0.001**
	**Model 3**		**Model 4**	
	**HR (95% CI)**	**p**	**HR (95% CI)**	**p**
**gout (yes)**	**5.772 (3.393, 9.820)**	** < 0.001**	**5.835 (3.517, 9.681)**	**0.001**
**Age (continuous)**	**1.107 (1.070, 1.144)**	** < 0.001**	**1.110 (1.076, 1.146)**	** < 0.001**
**Sex (female)**	**-**	**-**	**0.551 (0.310, 0.981)**	**0.043**
**RBP (continuous)**	**0.950 (0.925, 0.976)**	** < 0.001**	**0.953 (0.929, 0.978)**	** < 0.001**

*RBP* Retinol binding protein, *HR* Hazard ratio, *CI* Confidence interval. Model 1 and Model 3 were adjusted for age, gout, hypertension, ALB, Hb, and CT. Model 2 and Model 4 were fully adjusted. Binary RBP was analyzed in Model 1 and Model 2 while continuous RBP was included in Model 3 and Model 4. Model 1 and Model 3 were direct entry models while Model 2 and Model 4 were conditional stepwise forward models

**Table 3:** Comparison of Outcomes of all patients in our study

	Total (*n* = 480)	**RBP > 20.95 mg/L** (*n* = 368)	**RBP ≤ 20.95 mg/L** (*n* = 112)	*P*-value
Primary outcomes				
3-month mortality	**12 (2.5%)**	**5 (1.4%)**	**7 (6.3%)**	**0.011**
6-month mortality	**22 (4.6%)**	**12 (3.3%)**	**10 (8.9%)**	**0.012**
1-year mortality	**69 (14.4%)**	**42 (11.4%)**	**27 (24.1%)**	**0.001**
Secondary outcomes				
3-month independent walking rate	**113 (23.5%)**	**96 (26.1%)**	**17 (15.2%)**	**0.017**
6-month independent walking rate	**274 (57.1%)**	**223 (60.6%)**	**51 (45.5%)**	**0.005**
1-year independent walking rate	**339 (70.6%)**	**270 (73.4%)**	**69 (61.6%)**	**0.017**
Hospital stays (days)	**15.83 ± 7.37**	**16.01 ± 7.79**	**15.25 ± 5.80**	**0.502**
Hospitalization costs (CNY)	**78,493.20 ± 24,466.96**	**78,513.18 ± 24,526.43**	**78,427.53 ± 24,380.03**	**0.884**

**Table 4:** Logistics regression for mortalities and free walking abilities at 3 months, 6 months, and 1 year

	**RBP (≤ 20.95 mg/L)**		**RBP (continuous)**	
	**OR (95% CI)**	**p**	**OR (95% CI)**	**p**
**3-months mortality**	**4.088 (1.244, 13.436)**	**0.02**	**0.904 (0.836, 0.977)**	**0.011**
**6-months mortality**	**2.477 (1.019, 6.022)**	**0.045**	**0.935 (0.885, 0.987)**	**0.015**
**1-year mortality**	**2.168 (1.218, 3.859)**	**0.009**	**0.967 (0.939, 0.997)**	**0.03**
**3-months free walking ability**	**0.539 (0.296, 0.983)**	**0.044**	**-**	**-**
**6-month free walking ability**	**-**	**-**	**-**	**-**
**1-year free walking ability**	**-**	**-**	**-**	**-**

*RBP* Retinol binding protein, *OR* Odds ratio, *CI* Confidence interval

## Discussion

IN this study, we explored the relationship between RBP and the outcomes of hip fractures. As shown in the results, RBP might be a protective factor for the rehabilitation of patients who underwent hip surgeries due to hip fractures: patients with increasing RBP may face a low risk of mortality and favorable walking ability. Moreover, we also determined a cutoff point of RBP (20.95 mg/L) and the patients with high levels of RBP (> 20.95 mg/L) may also face a better prognosis.

We set relatively strict inclusion criteria. As we know, RBP is synthesized by the liver, so liver diseases may influence the levels of RBP, and then the RBP might be sensitive to liver status, instead of bone metabolism and nutrition. That is why we excluded the patients with severe liver disease. To better show the difference in RBP in patients with different outcomes, the PSM was carried out to reduce the impact caused by co-factors, and the ROC curve and the identification of cutoff points were also performed based on the matched populations. We believe that we can minimize the bias in this way. Similarly, the outcomes compared directly in groups of low and high levels of RBP may also face the impact of co-variables. Therefore, the logistic analysis was used to provide stronger evidence. As shown in Tables 3 and 4, the predictive value of high levels of RBP is not significant in the logistics models for 6-month walking ability and 1-year walking ability while in the univariate analysis, the 6-months and 1-year walking ability are significantly high in the high RBP group, which means the covariable may affect the waking ability. Therefore, we finally concluded that the low RBP group may have poor mortality at 3 months, 6 months, and 1 year, and free walking ability at 3 months.

Recent studies had reported the relationship between RBP and bone: RBP may associate with BMD and osteoporosis via multiple pathways. Li G and their team explored the impact of RBP1 on bone regeneration: they found that the RBP1 may promote osteogenic differentiation of bone marrow-derived mesenchymal stem cells through inhibiting RXRα-induced β-catenin degradation and then affect the process of bone regeneration [16]. Moreover, the pathway between PTH and RBP was also reported: PTH, PTH-related peptide, and (Bu)2cAMP increased the RBP mRNA level in chondrocyte cultures, and the PTH may regulate the bone metabolism by modulating RBP [17]. Otherwise, RBP may also impact bone metabolism through fat mass. It is reported that the rats with knockout RBP faced a decreased level of non-esterified fatty acids [18], and many studies also proved that the high RBP was associated with adipose tissue [19]. The expression of signal factors in adipose tissue, such as adiponectin, visfatin, and leptin, may affect BMD [20, 21]. It was also reported that the RBP as one of the adipokines, may interact with other adipokines including fibroblast growth factor 21 (FGF21), bone morphogenetic protein (BMP)-4, BMP-7, and so on, and these signaling factors were key factors in pathways involved in bone metabolism [22].

Population study also showed consistent results: a study enrolled 355 patients grouped by different levels of BMD showed that the RBP, as well as alkaline phosphatize, was positively correlated with BMD at the lumbar spine, femoral neck, and hip [13]. Moreover, a study comparing the predictive efficiency of various bone parameters reported that RBP, after alkaline phosphatase and age, was the strongest predictor for BMD in treated postmenopausal osteoporosis [23]. Similarly, many studies also suggested that RBP may relate to vitamin D by influencing nutrition status [24, 25]. As for the relation between RBP and fractures, a study that enrolled the patients with osteoporotic fractures and age-matched control subjects showed that the patients with osteoporotic hip fractures have lower RBP than their controls [26].

RBP plays an important role in metabolism, especially in adipose tissue [14]. The liver synthesizes the majority of RBP, but almost 20% of retinol was stored in adipose tissue [27]. The levels of RBP fluctuate rapidly and sensitively when the body faces different nutrition statuses: the RBP may increase facing malnutrition [28]. It was reported that the glucose transporter (GLUT4), a marker in adipocytes, was positively related to RBP [29]. A study enrolled the individuals who underwent gastric bypass surgery found that the patients with decreased body fat may have a significant reduction in RBP than those with unchanged body fat [30]. In a study about diet, the RBP decreased in the situation of a calorie diet while RBP in turn increased in the period of normal food [31]. In our study, the levels of RBP at the first time of hospital admission were selected to analyze, because RBP could sensitively indicate the nutrition status. Moreover, BMI and albumin as indicators of nutrition status were also analyzed in PSM, Cox models, and logistics models to control the bias caused by malnutrition.

There are some limitations to this study. First, our study is a retrospective single-center study based on relatively small samples, the missing data from follow-up and RBP may increase the bias. Moreover, the values of RBP are fluctuant. We cannot control the time from injury to admission. Though we select the data at the first laboratory test, the time may also affect the values. Thirdly, we failed to include some potential covariable, such as the diagnosis of osteoporosis and the use of anti-osteoporosis drugs. Lastly, walking ability as a functional outcome may be influenced by many factors, including the occurrence of complications and mechanical failure, fracture patterns, and so on. These factors not included in our study may cause bias in outcomes.

RBP as an indicator associated with bone metabolism and nutrition status could predict the outcomes of hip fracture. We could serve our patients better by noting the RBP and trying our best to improve their nutrition status, which may provide a better prognosis for patients. Moreover, due to the role played by RBP in many pathways, we may also notice the bone metabolism status of patients, and provide potential treatment. We hope experimental studies with a high level of evidence could prove the relation between RBP and outcomes of hip fracture.

## Conclusion

RBP may be associated with the survival and 3-month walking abilities of patients with hip fractures.

## Data Availability

The original contributions presented in the study are included in the article, further inquiries can be directed to the corresponding author.
